# Potential biological control of the pupal stage of the European grapevine moth *Lobesia botrana* by the entomopathogenic fungus *Beauveria pseudobassiana* in the winter season in Chile

**DOI:** 10.1186/s13104-019-4584-6

**Published:** 2019-08-28

**Authors:** Fabiola Altimira, Nathalia De La Barra, Patricia Rebufel, Sylvana Soto, Rodrigo Soto, Patricia Estay, Nancy Vitta, Eduardo Tapia

**Affiliations:** 1 Laboratory of Entomology, Instituto de Investigaciones Agropecuarias, INIA La Platina, Santa Rosa 11610, La Pintana, Santiago, Chile; 2 Laboratory of Phytopathology, Instituto de Investigaciones Agropecuarias, INIA La Platina, Santa Rosa 11610, La Pintana, Santiago, Chile; 3 Laboratory of Entomology, Servicio Agrícola Ganadero, Las Sophoras 120, Estación Central, Santiago, Chile

**Keywords:** *Lobesia botrana*, *Beauveria pseudobassiana*, Integrated pest management

## Abstract

**Objective:**

*Lobesia botrana,* the European grapevine moth, affects *Vitis vinifera* L. and other species of economic importance in a number of countries through damage caused by its larvae in berries and associated secondary diseases such as *Botrytis cinerea*. Control of the moth in urban areas is difficult due to poor chemical management of infested plants in houses. Additionally, in winter, *L. botrana* is in its pupal stage covered with a cocoon that prevents the penetration of chemical pesticides. For this reason, the objective of this work was to control the pupal stage with a formulation based on the entomopathogenic fungus *Beauveria pseudobassiana* in urban areas.

**Results:**

The strain RGM 1747 was identified as *B. pseudobassiana* by multilocus sequence analysis*.* The biocontrol activity of this formulated fungus against the infestation of vines with breeding pupae without cocoons showed 100% infection 21 days after inoculation under winter conditions. Finally, the biocontrol activity of the formulated fungus against natural infestations of *L. botrana* in winter in urban areas reached an efficacy of 51%. This result suggests that the *B. pseudobassiana* formulation is able to penetrate the cocoon and contributes to the integrated pest management of *L. botrana*.

## Introduction

*Lobesia botrana* (Denis and Schiffermüller) (Lepidoptera: Tortricidae), commonly known as the grapevine moth, occurs in Europe, Africa, Asia and in South America in countries such as Argentina and Chile [1], where the pest has been under the control of the Agricultural and Livestock Service (SAG, acronym in Spanish) since April 2008 [2]. The moth has spread to a wide range of regions in Chile from the Atacama region to the Araucanía region, causing disease in grapes (*Vitis vinifera*) and other plant hosts such as blueberry (*Vaccinium corymbosum*) and plum (*Prunus domestica*) [3]. The primary damage caused by this species occurs through larval feeding on berries, dehydration of tissue and fruit rotting, which leads to a subsequent round of fungal diseases such as grape mold (*Botrytis cinerea*) [4]. Currently, farmers have achieved effective chemical control and mating disruption of this pest, but reinfection of vineyards is caused by small urban outbreaks that are not controlled. Additionally, climate change contributes to shortening of its reproductive cycles and leads to appropriate conditions for new outbreaks within a season. As an alternative to reduce pesticide use to preserve our environment, health and nutrition, a goal is to use a formulation based on the entomopathogenic fungi (EPF) *Beauveria pseudobassiana* to control the pest in urban areas during its winter diapause.

## Main text

### Materials and methods

#### Biological material

All the pupae of *L. botrana* used for the in vitro and field trials were obtained from the breeding project of the Entomology Laboratory of INIA La Cruz. The strain *B. pseudobassiana* RGM 1747 was isolated from *Polistes gallicus* (Hymenoptera: Vespidae) and stored in the Bank of the Chilean Collection of Microbial Genetic Resources of INIA Quilamapu.

#### DNA extraction, PCR conditions and sequencing

The conidial DNA of strain RGM 1747 was extracted using the Quick DNA Fungal / Bacterial Kit (Zymo Research, CA, USA) following the manufacturer's instructions. The partial sequences amplified by PCR were for the *rpb1*, *rpb2*, *tef* and *Bloc* loci [5], which were sequenced at Macrogen (Seoul, South Korea). The DNA sequences determined for the four loci were submitted to the GENBANK Nucleotide Sequence Database under accession numbers MH048640, MH048641, MH048642 and MH048643 respectively.

#### Multilocus sequence analysis (MLSA) of *Beauveria* sp

Concatenated alignments were performed with MUSCLE for the *Bloc*, *tef*, *rpb1* and *rpb2* loci in 34 strains [6]. The evolutionary history was inferred using neighbor joining method [7] and the evolutionary distances were computed using the Tamura 3-parameter method [8]. The strength of the internal branches of the resulting trees was statistically evaluated by bootstrap analysis [9]. Finally, the evolutionary analyses were conducted in MEGA7 [10].

#### EPF formulation

The entomopathogenic fungi were spread over the surface of PDA (Difco ™, NJ, USA) in a plate. The fungi were grown for 1 week at 25 °C, and the spores and hyphae produced were collected through the addition of 10 mL of PBS (pH 7.4) containing 0.05% Tween 20 (Merck, Darmstadt, Germany) to a plate. The conidial concentration was determined by counting in a Neubauer chamber and adjusted to 10^6^ conidia/mL. The formulation was generated to maintain the fungal biomass in the winter using 2.5% rice flour, 2.5% skim milk, 20% glycerol (Merck), 0.2% Silwet L-77 Ag (Arysta LifeScience, Santiago, Chile), 73.8% distilled water and 1% EPF at 10^6^ conidia/mL. All the components were mixed in a beaker at 300 rpm for 30 min.

#### In vitro EPF formulation evaluation against *L. botrana*

The assessment of the formulation was carried out in in vitro assays at 25 °C. One milliliter of the formulation mix was placed in the center of a paper disk that covered the bottom of a plate, and ten moth pupae were deposited on the paper. As a control, water was used instead of the formulation. The infection of pupae with EPF was monitored every day for 8 days. The experiment included three replicates.

#### Field trial

The assay was developed in the *V. vinifera* ´Red Globe´ field at La Platina research station in the Metropolitan region of Santiago, Chile, in natural environmental conditions in July, during the winter. The field trial was arranged in a randomized block design with four treatments, each with four replications with three vines each (4 × 4 × 3). To infest the vines, ten pupae without cocoons per plant were deposited under the rhytidome, covered with a tulle mesh and fixed with adhesive tape to avoid dispersion of the pupae and the entry of other arthropods. The first treatment was performed using one application of the formulation. In the second treatment, we used two applications on days one and seven. In the third treatment, we performed applications during days one, seven and fourteen. The fourth treatment was the control using water. The EPF concentration for treatments one, two and three was 10^9^ CFU/L. The applications were performed with a hand pump, and wetting was performed with a volume of 500 mL per vine. Each treatment was inspected 7 days after each application. For the inspection of the infected pupae, we removed the pupae from under the rhytidome and deposited them in a plate containing a humid paper disk when the EPF had completed colonization. The evaluation of efficacy was performed over 48 h at 25 °C.

#### Urban trial

The assay was developed in *V. vinifera* plants located in urban residences in the Metropolitan region of Santiago, Chile, under natural environmental conditions in August, during the winter diapause of pupae with cocoons. The urban trial was arranged in a randomized block design with four treatments, each with three replications with three vines each (4 × 3 × 3). The distribution of the blocks in the urban area was set up according the captures of the moths in pheromone (E7, Z9-dodecadienyl acetate) traps during the spring and summer by SAG. The treatments and the methodology for the application of the formulation and inspection of the vines were the same as those described for the field trial. Finally, the pupae grown in cocoons with EPF were dissected to determine infection by the fungus. The pupae were dyed with lactophenol blue (Merck) to observe the penetration of the fungus into their structure.

Temperature and humidity data during the field and urban trials were collected through the weather station network (https://agromet.inia.cl/).

### Statistical analysis

The efficacy of the formulation in vitro and in field trials was determined for uniform populations [11], and the efficacy in the urban trials was determined for nonuniform populations [12]. The percentages of efficacy in the field test and urban test were compared by Tukey’s HSD test (α = 0.05). All the experiments were analyzed using the software Statgraphics Centurion XVII (Statpoint Technologies Inc., VA, USA).

### Results

#### Molecular species identification of an EPF isolate

**Fig. 1:**
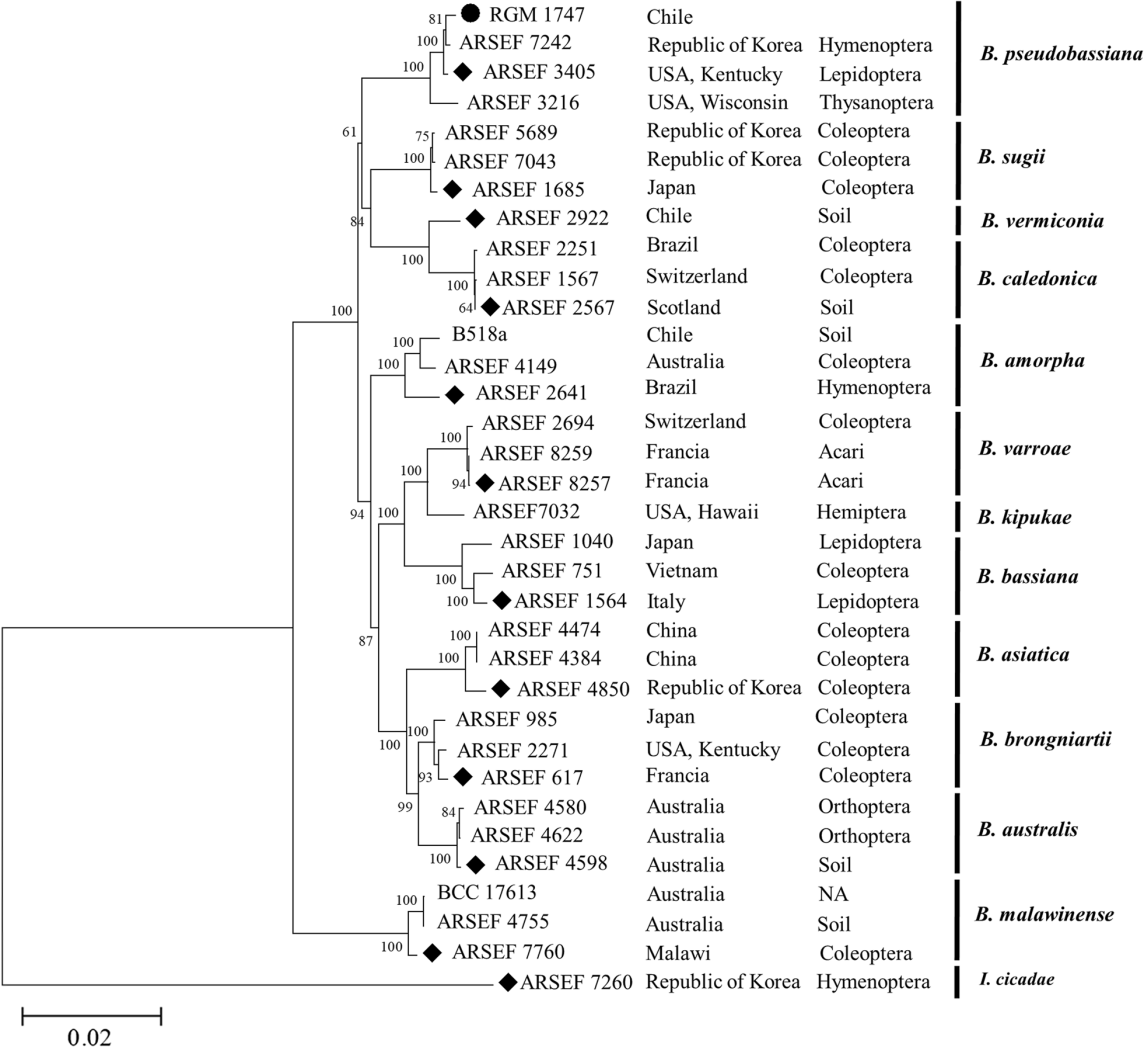
Phylogenetic tree of *Beauveria* strains based on multilocus analysis of *Bloc*, *tef*, *rpb1*, and *rpb2*. The evolutionary history was inferred using the neighbor-joining method. The evolutionary distances were computed using the Tamura 3-parameter method. Bootstrap values were over 50% (1000 replications). The tree is drawn to scale, with branch lengths measured in the number of substitutions per site. *I. cicadae* ARSEF 7260 was used as the outgroup. The strains associated with the type of material are indicated with diamonds, and our strain is indicated with a circle. *NA* not available

The *Beauveria* sp. RGM 1747 strain was subjected to molecular characterization using MLSA methodology, which indicated that this strain belongs to the clade of *Beauveria pseudobassiana*, as shown in Fig. 1 and Additional file 1: Table S1. It was grouped with other strains that have been isolated from different insect orders and countries such as Hymenoptera in South Korea and Lepidoptera and Thysanoptera in the USA.

#### In vitro evaluation

**Fig. 2:**
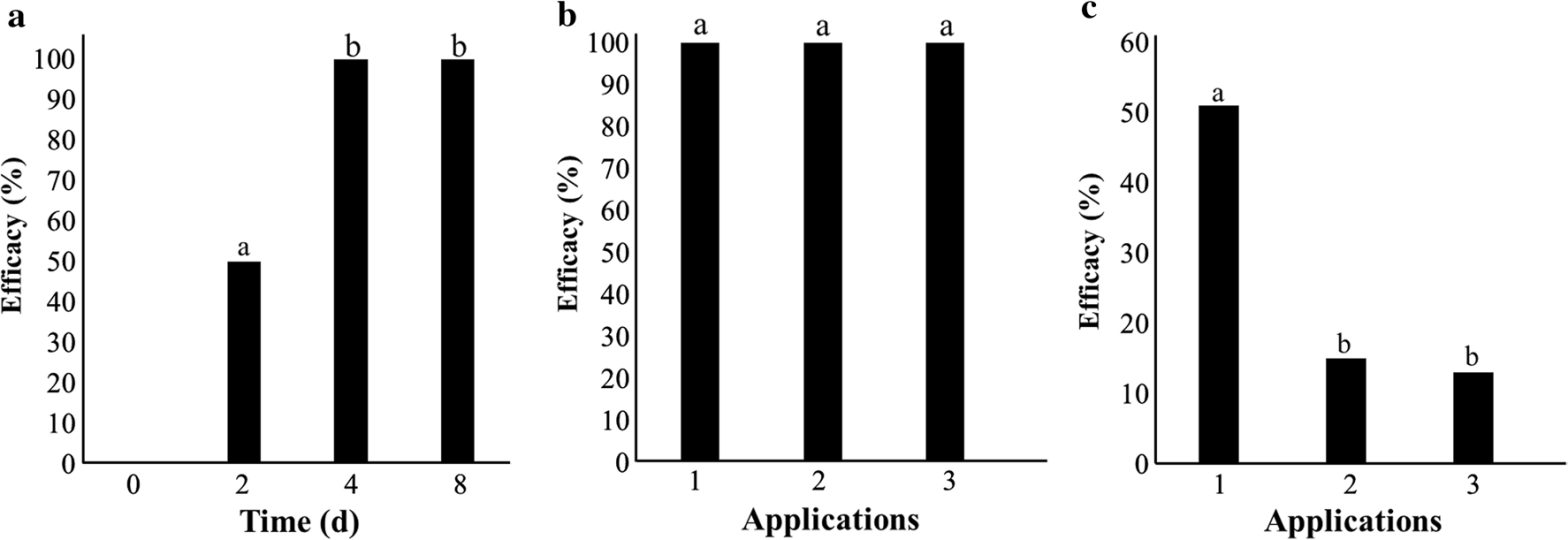
Efficacy of experiments in the laboratory, field and urban areas. **a** Efficacy of the laboratory application in the pupae over 8 days. **b** Efficacy of the field application of the EPF formulation on plants with controlled infestations. **c** Efficacy of urban applications of the EPF formulation on infested vines in gardens. In both **b** and **c** the treatments were as follows: the first treatment was performed with one application on day one. Treatment two was performed with two applications: one on day one and one on day seven. Treatment three was performed with three applications: on days 1, 7 and 14. Each treatment was inspected 7 days after each application. The evaluation of efficacy was performed after 48 h at 25 °C. The in vitro and field efficacy results were calculated with the method of Abbott. The efficacy results for the urban assay were calculated with the method of Henderson and Tilton. The same letters in each bar chart indicate that there was no statistically significant difference by Tukey’s HSD test (α = 0.05)

Once the EPF formulation was prepared, in vitro infestation was carried out at 25 °C under high humidity. All the pupae were infected by the EPF with 100% efficacy by four dpa (Fig. 2a).

#### Field trial evaluation

The vineyard infestations were performed under controlled conditions with an average temperature of 9.1 °C and relative humidity of 78.3%. In the three treatments with the application of the EPF formulation, the development of incipient mycelia and conidia of *B. pseudobassiana* in different areas on the head and sutures of the pupa was observed. All the examined pupae were infected by *B. pseudobassiana,* which showed the capacity of the EPF to achieve colonization in winter conditions with an efficacy of 100% at 21 dpa (Fig. 2b).

#### Urban trial evaluation

**Fig. 3:**
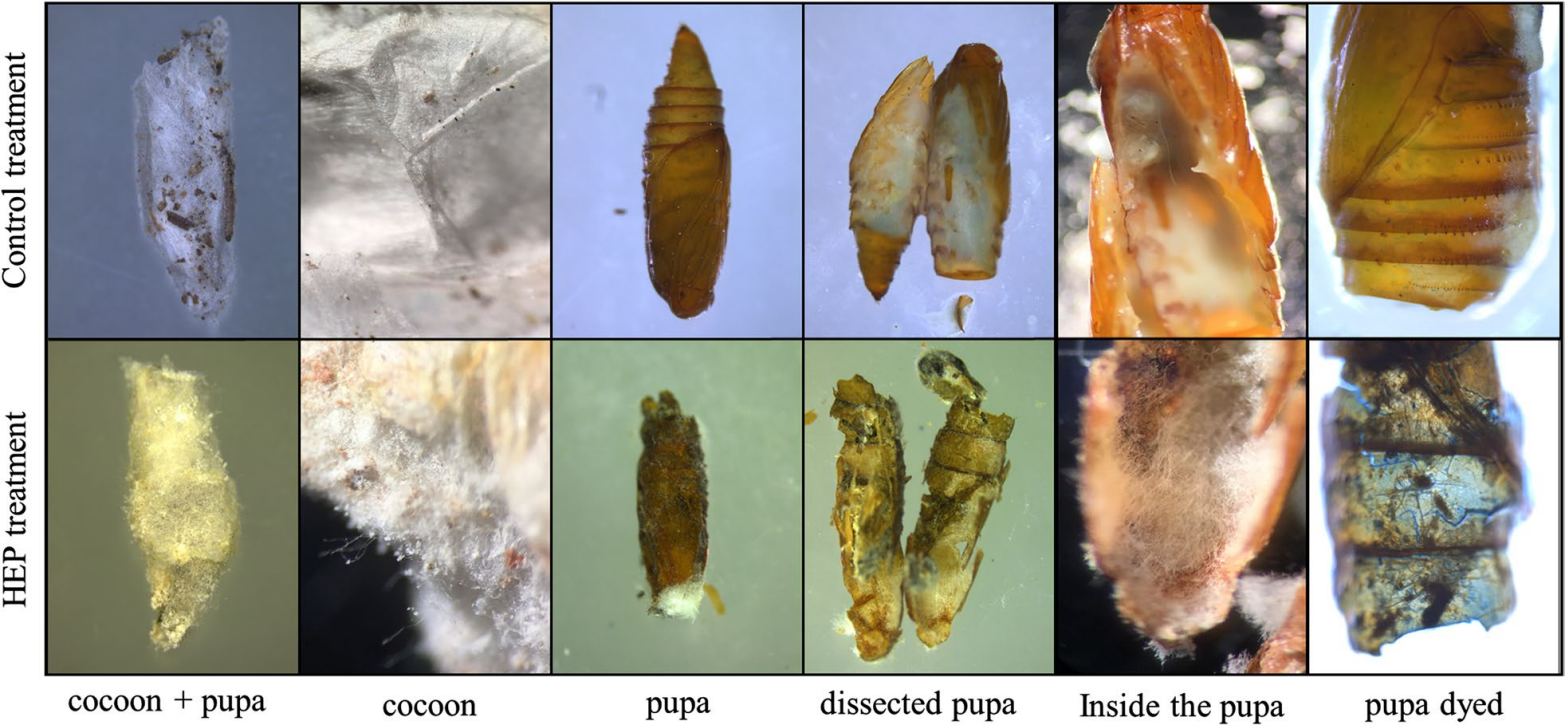
Representative images of EPF colonization on pupae of *Lobesia botrana*. In the first row, images of a pupa subjected to the control treatment are shown. The lower row shows images of a pupa infected through EPF formulation treatment in the urban sector. The hyphae that penetrated the cuticle of the pupa are stained with lactophenol blue

We used the same formulation treatment as in the field trial, but the infestations of *L. botrana* were natural in this case. The average temperature and relative humidity in the assay were 8.4 °C and 73.7%, respectively. In these assays, not all the examined pupae were infected by *B. pseudobassiana*. The efficacy reached 51% in the first treatment (Fig. 2c). The efficacy in the second and third treatments reached 15% and 13%, respectively. The pupae colonized by the EPF showed development of different levels mycelia and conidia of *B. pseudobassiana* in several areas on the head and sutures of the pupa when the cocoon was removed (Fig. 3).

### Discussion

Based on the increasing interest in biological control, we identified and classified *B. pseudobassiana* RGM 1747 and compared its activity with that of other isolates from EPF-infected insects around the world that had already been sequenced (Fig. 1). In Chile, the strain RGM 1747 was isolated from *P. gallicus* and showed activity against *L. botrana* in this work. Additionally, Gerding et al. reported that this EPF showed no negative effect in bees and found that the fungus achieved control over *V. germanica* [13]. Furthermore, the use of biological products in winter stages reduces the possibility of cross-activity with beneficial arthropods due to a decrease in their activity during winter because of low temperatures and a lack of flowers. In Chile, there are no chemical and biological products for controlling the pupae during the winter. Therefore, the application of EPF in winter could be compatible with application of fungicides in spring within an IPM program for fungal disease control in grapes.

In the study of Cozzi et al. [14] determined the mortality of 6 EPF isolates on *L. botrana* larvae in vitro assay. The best strain, *B. bassiana* ITM 1559, showed a 55% mortality. Additionally, in field assays the incidence of bunches injured by *L. botrana* larvae were significantly reduced by this strain treatment, compared to the untreated control. In our study, the in vitro formulation evaluation achieved 100% efficacy in *L. botrana* due to the occurrence of all the conditions required for the EPF to colonize the pupae. However, we considered it necessary to perform field trials under the temperature and humidity conditions of July. This trial reached an efficacy of 100% and was considered representative of the behavior of the fungi because under the environmental conditions and oscillations in temperature and humidity that occurred in this trial.

On the basis of the results of the field trial, we assessed an assay conducted in urban zones during August. The primary barrier to the EPF is the cocoon, which provides natural protection against pathogens and arthropods. In this case, the efficacy reached 51% using only one application. The pupae from the urban zones showed hypha and conidia on the cocoons and between the sutures of the pupae. This result demonstrated the ability of the fungus to penetrate the cocoon and colonize the pupa (Fig. 3). The other treatments in which we performed two and three applications did not show significant differences (15% and 13% efficacy, respectively). In addition to the decrease in efficacy caused by the cocoon in all the treatments, another possible explanation for this difference is that during the first application, the EPF consumes most of the carbon sources and proteins from the pupae and the formulation, but after seven dpa, opportunistic fungi such as *Aspergillus* sp. *Penicillium* sp. and *Rhizopus* sp*.* begin to appear (observations of urban samples). These fungi may have taken advantage of the components of the formulation and reduce the efficacy of the product.

In conclusion, we achieved an efficiency of 51% with one application of EPF formulation in urban areas to control the pest during its winter diapause. Additionally, the formulation is useful in fruit crops because pollinators and antifungal chemical applications are not encountered in the field. Finally, possible annual applications in urban and rural zones contribute to the control of *L. botrana* in the country in combination with other IPM strategies.

## Limitations

To improve the formulation, it will be necessary to examine its components and different concentrations thereof to increase their efficacy. We will need to perform a series of urban trials over time to optimize the use of the EPF.

## Supplementary information

**Additional file 1: Table S1.** The table shows the partial sequences *Bloc*, *tef*, *rpb1* and *rpb2* along with their access numbers plus the percentages of identity and coverage. These sequences were used to perform the MLSA.

## Data Availability

All amplified DNA fragments from the *Beauveria* isolate were registered in NCBI GenBank and are available in Additional file 1: Table S1. The isolate of *B. pseudobassiana* was deposited in the Chilean Collection of Microbial Genetic Resources, International Depository Authority, with the Access Number RGM 1747.

## Abbreviations

CFU: colony-forming units
IPM: integrated pest management
dpa: days post application
EPF: entomopathogenic fungus
