# Supplementary material for: Potential biological control of the pupal stage of the European grapevine moth Lobesia botrana by the entomopathogenic fungus Beauveria pseudobassiana in the winter season in Chile
Source: BMC Res Notes. 2019 Aug 28;12:548. doi: 10.1186/s13104-019-4584-6 (PMC6714376; doi:10.1186/s13104-019-4584-6)
Supplement: Supplementary file 1 — Additional file 1: Table S1. The table shows the partial sequences Bloc, tef, rpb1 and rpb2 along with their access numbers plus the percentages of identity and coverage. These sequences were used to perform the MLSA. [file 13104_2019_4584_MOESM1_ESM.docx]

**Table S1** Comparation between molecular marker sequences of *B. pseudobassiana* RGM 1747 and the type strain *B. pseudobassiana* ARSEF 3405

| Molecular marker | GenBank molecular number | | Identity (%) | Coverage (%) |
| --- | --- | --- | --- | --- |
|  | strain RGM 1747 | strain ARSEF 3405 |  |  |
| Bloc | MH048640 | HQ880723 | 99 | 100 |
| *tef* | MH048641 | AY531931 | 99 | 100 |
| *rpb1* | MH048642 | HQ880723 | 99 | 93 |
| *rpb2* | MH048643 | HQ880936 | 99 | 94 |
